# Need to Knowledge (NtK) Model: an evidence-based framework for generating technological innovations with socio-economic impacts

**DOI:** 10.1186/1748-5908-8-21

**Published:** 2013-02-15

**Authors:** Jennifer L Flagg, Joseph P Lane, Michelle M Lockett

**Affiliations:** 1 Center for Assistive Technology, University at Buffalo (SUNY), 100 Sylvan Parkway, Suite 400, Amherst, NY, 14228, USA

**Keywords:** Knowledge translation, Technology transfer, Commercial transaction, Evidence-based, Technology-based, Scientific research, Engineering development, Industrial production, States of knowledge, Innovation

## Abstract

**Background:**

Traditional government policies suggest that upstream investment in scientific research is necessary and sufficient to generate technological innovations. The expected downstream beneficial socio-economic impacts are presumed to occur through non-government market mechanisms. However, there is little quantitative evidence for such a direct and formulaic relationship between public investment at the input end and marketplace benefits at the impact end. Instead, the literature demonstrates that the technological innovation process involves a complex interaction between multiple sectors, methods, and stakeholders.

**Discussion:**

The authors theorize that accomplishing the full process of technological innovation in a deliberate and systematic manner requires an operational-level model encompassing three underlying methods, each designed to generate knowledge outputs in different states: scientific research generates conceptual discoveries; engineering development generates prototype inventions; and industrial production generates commercial innovations. Given the critical roles of engineering and business, the entire innovation process should continuously consider the practical requirements and constraints of the commercial marketplace.

The Need to Knowledge (NtK) Model encompasses the activities required to successfully generate innovations, along with associated strategies for effectively communicating knowledge outputs in all three states to the various stakeholders involved. It is intentionally grounded in evidence drawn from academic analysis to facilitate objective and quantitative scrutiny, and industry best practices to enable practical application.

**Summary:**

The Need to Knowledge (NtK) Model offers a practical, market-oriented approach that avoids the gaps, constraints and inefficiencies inherent in undirected activities and disconnected sectors. The NtK Model is a means to realizing increased returns on public investments in those science and technology programs expressly intended to generate beneficial socio-economic impacts.

## Background

### Generating technological innovations in publicly funded R&D programs

Governments and societies are equally enthralled with technological innovation as a panacea for improving the quality of life in domestic society and for competing economically in a global marketplace. To achieve this end, numerous government programs fund scientific research and engineering development projects, with the expressed intention to generate technology-based innovations that are expected to result in beneficial socio-economic impacts. As economies and budgets contract, and sponsored grantees are tasked with demonstrating outcomes and impacts, there is greater interest in exploring evidence-based approaches to accomplishing technological innovation.

### Outgoing linear model of innovation

Within this system, despite decades of investigation on how to innovate successfully, the so-called linear model remains the dominant paradigm. That is, government allocates funding to scientific research, which somehow yields socio-economic benefits. However, the linear model has been largely discredited [1-4], specifically because it overstates the importance of research at the expense of downstream development and production activities.

The model persists in policy and practice because free market economic systems avoid investing public funds in private enterprises, thereby making the academic and non-profit sectors the default recipients. This circuitous flow of resources leaves industry as the passive recipient of research publications and development patents supplied by the sponsored programs, which then require private investment to transform them into commercial goods and services [5,6].

The linear model’s main impediment to innovation is the presumption that all projects must commence with new scientific research, with little consideration of either its necessity or its likely contribution to the expected outcomes. The ‘fuzzy front end’ of successful innovation projects requires effective need identification and market scoping [7,8]. A lack of adequate market information or cost considerations is clearly associated with project failure [9,10].

However, the scholars who receive public funding often lack the training to value or conduct these essential activities, which are either ignored or poorly performed. Rather, the academic training, culture and incentives focus on conducting research and publishing the results, regardless of the intended innovation’s requirements for new fundamental knowledge or its eventual contribution to commercial innovations.

### Incoming collaborative models of innovation

Sponsored programs charged with generating technological innovations seek models and methods that effectively and efficiently result in outputs capable of industry uptake and commercialization. Two U.S. programs – the Small Business Innovation Research (SBIR) program, and the former Advanced Technology Program (ATP) – were established with this mission, yet both have fallen short of that goal [11,12]. The European Union is also challenged by efforts to transform science into socio-economic benefits through supply-push approaches, and is instead now encouraging investigators to consider demand-pull commercialization issues as early as the proposal stage [13]. Because of these problems, some agencies are infusing calls for new approaches to knowledge translation and implementation in their solicitations. For example, the U.S. National Institutes for Health added language to their 2012 funding announcements calling for projects to address the discovery to delivery gap through more intense collaboration between sectors [14]. Similarly, the U.S. Department of Education established a multi-year national center – operated by this paper’s authors – to integrate knowledge translation with technology transfer processes, in an effort to improve the yield from sponsored programs intending to generate technological innovations with beneficial socio-economic impacts [15].

As the host institutions for most publicly funded projects in this area, universities established technology transfer offices (TTO) to broker the transition of knowledge from the laboratory to the marketplace. However, the TTOs face the same constraints imposed by a supply push model, where technical answers are in search of a market question [16,17]. The problem’s persistence is evidenced by the range of efforts introduced to address it. For example, University Innovation Centers and Proof of Concept Centers have been established to act as liaisons between academics and industry [18,19], while Technology and Innovation Centers and Collaborative Innovation Centers attempt to address innovation challenges on a regional level [20]. However, these organizations have not yet found a model that is globally applicable to a majority of their efforts. In fact, Holly’s [21] call for the identification of broadly applicable models to accelerate innovation outcomes from sponsored university programs remains valid.

### Industry generates and requires commercial innovations

Corporations transform scientific and technical knowledge into innovative commercial products and services – they profit or perish. Over time, industry has established ‘best practices’ in product development encompassing essential activities (stages) and critical decisions (gates), which are codified in the Product Development and Management Association’s (PDMA) series of handbooks and tool books [22]. These best practices are taught in vocational and business schools so that all participants along the product value chain can anticipate, plan and execute the proper methods in the proper sequence to deliver the intended innovations and generate the expected profits.

The product development models traditionally assume that all activity occurs within the corporate entity, where all resources are internal. Few consider the requirements of a process where one sector (academia) is funded to conduct the scientific research, while others are expected to transform the resulting knowledge into commercial innovations. The existing models do not differentiate between methodologies (*e.g*., scientific research, engineering development, industrial production), nor the requirements of their respective knowledge outputs (*e.g*., conceptual discoveries, prototype inventions, commercial innovations). Furthermore, existing models do not consider the importance of leveraging specific forms of communication to share these different knowledge outputs with stakeholders in diverse sectors.

Management literature addresses the persistent barriers between R&D and marketing personnel by stressing the importance of coordination and cross-functional teams to new product success [23-26]. Similar barriers require effective communication strategies when information must be shared between multiple sectors with different training, cultures and values [27-29]. However, even recent literature on open innovation focuses on business-to-business interactions, rather than cross-sector collaborations between academia and industry [30].

### Overcoming the discontinuity in technological innovation

Government policies direct funding for ‘R&D’ in the public and non-profit sectors, yet require industry best practices to generate market innovations. This discontinuity requires an intervention strategy that accommodates the constraints on the former while applying the capabilities of the latter. In response to this need, Lane and Flagg articulated the technological innovation process as consisting of three distinct methodologies: scientific research, engineering development, and industrial production [31]. Each methodology generates knowledge outputs in a unique state: respectively, conceptual discovery, prototype invention, and commercial innovation. Extending these concepts, Stone and Lane subsequently applied a logic model to describe how to reconcile the need for rigor inherent in the three methods underlying technological innovation, with the need for relevance in order for their outputs to achieve commercial success in the competitive marketplace [32].

While manufacturing represents later stages of a systematic innovation model, the manufacturer’s constraints and capabilities must be recognized and integrated into decision criteria under the upstream research and development phases [33]. For internal or closed innovation, this is taken as a given for the project to advance through management decision gates. However, for external or open innovation, the organization developing a solution to a problem must ensure that the preliminary R&D work will meet the manufacturing partner’s internal standards for rigor and relevance through active and ongoing communication [28]. In this case, the manufacturer is the knowledge producer’s customer, and their needs – rather than just the needs of a product end user – are paramount to success. Failing to recognize the manufacturer as the customer for research and development project outputs can have the disastrous consequence of the target company simply declining to invest their own resources in advancing the project toward a commercial innovation.

Unfortunately, the theories and models of innovation published in academic literature are too abstract to be applied by industry practitioners [34,35]. If innovation and new product development practitioners have difficulty transforming abstract concepts into working applied models, then academics from outside of the business and management realm face even greater challenges in the translation and application of business concepts and terminology. We assert that the barrier to increasing the yield from sponsored innovation programs is not a lack of theoretical constructs, but a lack of operational guidelines.

Therefore, it is of particular importance that sector-related jargon is transformed into a language and format that can be appreciated by academic researchers who receive funding from programs that are intended to generate technological innovations [36]. The authors theorize that a plain-language operational-level (step-by-step) model that integrates best practices from new product development and innovation literature, combined with evidence-based knowledge communication strategies, has the xpotential to improve the success of the nation’s publicly funded innovation programs. Such a model could significantly improve the researchers’ ability to apply those practices so that they can then more efficiently and effectively span the language, culture and practices of government, academic and industry sectors, to anticipate the opportunities and constraints of their downstream partners, to collectively achieve the intended outcomes and impacts.

## Discussion

### A Need-Driven rather than Actor-Driven Perspective

The ‘Need to Knowledge’ (NtK) Model embodies the methods considered to be most appropriate by the respective professions by combining three related sets of best practices. First, the PDMA handbooks and tool books provided the majority of details needed to create an operational-level version of the engineering development and industrial production phases of the innovation process, while Campbell and Stanley’s work on research design [37] informed the activity steps in the scientific research phase. Finally, Graham and colleague’s [38] work on the Knowledge to Action model offered the activities required to effectively communicate knowledge to different stakeholder groups for implementation. The review of these works resulted in the integration of nearly 60 action-oriented steps into a stage/gate framework, as well as 70 tips related to the effective completion of steps.

The source content has been stratified within and across the three methodological phases of scientific research, engineering development, and industrial production, punctuated by analysis and evaluation at the decision gates. Collectively, the elements comprise a stage/gate model specifically designed for publicly funded research and development organizations who require cross-sector collaboration to generate successful technology-based innovations.

**Figure 1:**
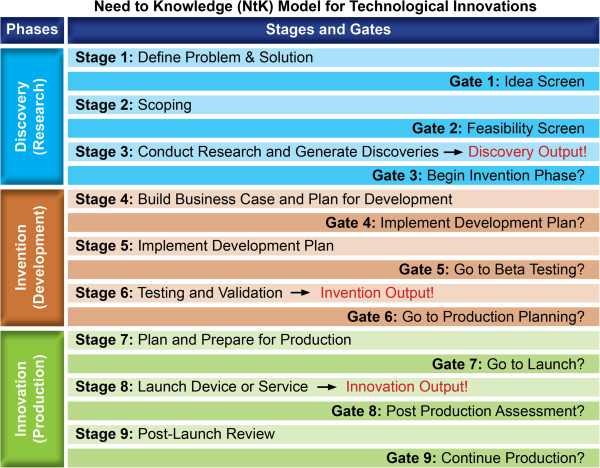
NtK Model phases, stages, gates and outputs.

Figure 1 shows a simplified version of the NtK, displaying the three phases in the left column. The right column shows the three stages and three gates within each phase, as well as each phase’s output. This output becomes input to the subsequent phase. This simple framework captures the entire technology-based innovation process from need identification to innovation deployments.

A scoping review of academic and industry literature on technological innovation yielded 230 relevant articles, from which about 1,400 salient points were excerpted and then interjected as supporting evidence for specific stages, gates or activities within the NtK Model. Narrative excerpts were preserved as exact quotations when possible, or paraphrased where necessary to provide the appropriate context. Narrative excerpts were designated as ‘primary’ when they were drawn directly from the cited articles, or designated as ‘secondary’ when the article was paraphrasing other sources. All primary and secondary narrative excerpts – along with citation information such as article authors, title, journal name and volume/issue – were catalogued through an online entry form that connected the excerpts with the NtK Model. For example, as an excerpt was entered into the form and associated with a step, a hyperlink for ‘supporting evidence’ would appear next to that specific step in the NtK Model. The hyperlink contains the narrative excerpt along with the full citation. See Additional file 1 for supplementary detail regarding the NtK Model [39-43].

### Nine activity stages and nine decision gates: an example

The following section describes the methods, activity stages, and decision gates in greater detail, combined with an example drawn from the author’s prior experience to demonstrate the operability of the NtK Model across all three methodological phases. This example is written from the perspective of a broker engaged in transferring a prototype invention (automatic jar opener), to an international manufacturer for commercialization as an assistive technology device to assist persons with disabilities and the elderly.

### Phase I: research activity

It is important to note that the NtK Model does not assume that every project begins with a research project, even though the funding for R&D is typically channeled through universities. Instead, research activity is considered as an option only after due diligence has been performed in Stage 1 to first identify a need and propose a solution, and after Stage 2, where the solution is vetted for feasibility. Both Stages 1 and 2 are ultimately necessary to optimize the chance for a new project to result in a commercial innovation [44-48]. Stages 1 and 2 are a critical departure from the ‘fund science first’ linear model, which overemphasizes rigorous research designs at the expense of output relevance to technological, market and business constraints.

#### Stage 1: define problem and solution

A government sponsored invention broker identified a functional need and business opportunity for an automated jar opener [49]. A review of existing products showed them to be inadequate to meet the needs of persons with disabilities and the elderly, nor had they been designed and marketed to meet the needs of mainstream markets such as children and multi-tasking adults. The combination of niche and mainstream markets, and the presence of sub-optimal product offerings passed the Gate 1 Idea Screen for a potential product solution to address a significant functional problem.

#### Stage 2: scoping

Preliminary feasibility assessments included a search of the current marketplace via web and catalog searches, phone calls to companies, and a scan of the U.S. Patent and Trademark Office’s Patent database. They revealed that a working prototype has recently won a national invention competition. The inventor had subsequently assigned the intellectual property rights to the corporation sponsoring the competition. Further investigation revealed that the company had not forwarded the invention to internal engineering development because of dissatisfaction with the prototype’s capabilities and their estimates of a small and niche market for a future product.

To assess the company’s position, the broker conducted a panel discussion with potential product customers, which suggested that the market was broader than anticipated. The primary targets were home and professional cooks, but the latent market also included a growing segment of adults with a wide range of functional limitations in grasping and twisting mechanics, such as people with limited strength, reduced sensation, and painful joints – particularly older persons with arthritis. The broker conducted in-depth market, consumer and technical analyses rigorous enough to meet industry standards, which justified continuing the project beyond Gate 2 – the Feasibility Screen.

Following Stages 1 and 2, the NtK model asks project managers to consider if they require any additional fundamental knowledge beyond what already exists to pursue the proposed solution. Instead of commencing a new research study from scratch, an innovation project may first look to the global base of existing knowledge, such as publication databases and patent repositories. If the necessary knowledge already exists in a valid and reliable form, the project may be able to bypass the scientific research stage and thereby save both time and money. However, the project may indeed have to design and conduct a research study to reconcile conflicting findings in the literature, or to simply fill a gap in existing knowledge. The critical distinction between the NtK Model and the linear model is that the former treats research as an optional step while the latter assumes all projects require and therefore commence with research.

#### Stage 3: conduct research

The project considered the need to design and conduct research to generate new fundamental knowledge. The team determined that all required scientific knowledge was available in the existing literature base, and the conceptual knowledge was already embodied in a proof-of-concept prototype. Some technical details were yet to be finalized. For example, this project required an understanding of how best to grasp a jar while mechanically breaking its vacuum seal. However, this type of exploration is more closely associated with technical development work because it could only occur after the project had received a green light to progress through development activities; it had to be preceded by the acquisition of consumer input; and it involved physical testing of prototype mechanics. Therefore, the team articulated the relevant conceptual discoveries from the scientific literature and proceeded directly to Stage 4. Of course, they reserved the right to conduct research if deemed necessary to advance through a subsequent Stage.

### Phase II: development activity

Research has demonstrated that too narrow a focus on technology at the expense of the business case or market potential of a product has negative consequences for project success [33]. As such, Stage 4 of the example demonstrates how input from marketing and sales experts can be used to guide the technology development activities of engineers, while Stage 5 shows the importance of including manufacturing personnel in this stage [50].

#### Stage 4: build business case and establish development plan

Given that an international appliance manufacturing corporation held the intellectual property rights for the invention, they were the ideal co-development partner for this project. This obviated a search for an appropriate corporate partner. However, this company was hesitant to allocate resources to development of the jar opener without additional market information — a significant potential barrier that would have doomed the project internally. Instead, the knowledge broker generated a preliminary business case to outline potential target market segments and make annual sales projections for the envisioned product. The corporation reviewed this analysis and decided to proceed. The intellectual property considerations were explicitly detailed, with both parties agreeing that the broker would only provide suggestions for functions and features. This gave the corporation full freedom and flexibility to determine how best to incorporate those suggestions into the product, thereby eliminating questions about design ownership, and allowing all rights to solely remain with the corporation. At this time, all parties’ roles were clearly agreed upon so that each contributor was aware of what was required of them and when they could anticipate execution of their duties.

In order to determine the highest priority functions and features of the jar opener, the knowledge broker conducted a series of three consumer focus groups. The groups applied industry standard methodology (*e.g*., three groups, 12–15 persons per group, purposive sampling, trained moderator, scripted process) to ensure that the corporate partner would view the results as valid and reliable. The groups yielded detailed suggestions related to the look and function of the device, which were provided to corporate engineers. Based on overwhelming positive feedback received from focus group participants, the business case was deemed to be valid, thereby easily allowing the project to pass through Gate 4.

#### Stage 5: implement development plan

The broker generated a list of 29 key product functions and features. The company’s first beta-level prototype incorporated most of the recommendations. Several were considered cost prohibitive in a first generation product, but were held in reserve in case profits were sufficient to justify a second generation version in the future. One great advantage over competing internal product proposals was that this design incorporated a motor that already existed as surplus stock from a discontinued product line. No doubt, the ability to incorporate a supply of over one half million motors benefitted the initial cost and the return on investment calculations. At Gate 5, the broker determined that the corporation had successfully integrated the critical user requirements, while the cost savings from the surplus motors helped gain a positive decision from corporate management. As a result, the project moved forward into Stage 6.

#### Stage 6: testing and validation

The broker recruited participants for two beta focus groups from a sample of the alpha focus group participants. The corporation generated a functioning proof of concept prototype as well as three static foam models for testing by these individuals. The models allowed participants to react to and comment on the way their previous recommendations from the alpha focus group were integrated into the product. Input was specifically sought on handle placement and configuration, and size and placement of the device’s activation button. All consumer design input, purchase intent, and price point information was again forwarded for consideration by the company. Gate 6 asked the collaborating organizations to determine if the prototype invention demonstrated sufficient profit to the company and utility to the target customers. All agreed to proceed to the Production Phase.

### Phase III: production activity

Functional prototypes prove a concept but require commercial hardening to prepare for large-scale manufacturing while ensuring quality control. The resulting products require distribution, sales and support in the competitive marketplace. The manufacturing and marketing involved represent the business practices of private corporations, which minimize the risks and maximize the returns from commercial innovations.

#### Stage 7: production planning and preparation

All materials specification, tooling design, and production planning was completed by the company and their subcontractors. The company initiated an internet-based roll-out of the product during the fall, using an initial production run to gauge consumer interest during the holiday season prior to committing to a production run as a full-fledged product, along with the cost of distribution and stocking at retail outlets. At the price point originally identified through focus groups ($39.99), the initial production run sold out in weeks. At Gate 7, encouraging sales at the selected price point dictated that the project moved forward toward a full-scale launch.

#### Stage 8: product launch

Once inventories were replenished, the company introduced the product through mainstream retail stores while continuing internet sales. First year sales topped one million units. Production and design refinement were ongoing. Continuous monitoring of consumer feedback, a noted success factor in new product development [44], informed the product’s future. Gate 8, Post-Production Assessment, led to the introduction of multiple versions of the automatic jar opener, including new features (*e.g*., can and bottle openers), different designs (*e.g*., slimmer, multi-material), as well as various activation mechanisms.

#### Stage 9: post-launch review

Perhaps one of the most neglected stages of activity for both industry and academia alike calls for improved monitoring to determine the actual social and economic impacts of sponsored projects. A summary of evaluations for the Advanced Technology Program suggested that future evaluation programs should include retrospective analyses based upon market data, and should pursue retrospective analyses of failures as well as successes [51]. In this example, where a private corporation’s survival depends on product success in the market, the manufacturer carefully tracked the product line’s lifecycle. In parallel, the broker assessed the impact of the automatic jar opener on the community of persons with disabilities by conducting an efficacy study. The study demonstrated that the new jar opener device was indeed useful to persons with a range of physical limitations, including arthritis, carpal tunnel symptoms, hemiplegia, and muscle weakness, and was proven to be more useful than the competing products available in the marketplace at the time of introduction [52]. The study results were of interest to the future product planning of the partner corporation, as well as to the government agency sponsoring the knowledge broker.

### Moving knowledge between phases and sectors

Industry best practices assume that one organization – typically a private corporation – manages the entire innovation process, which is initiated in response to an opportunity to satisfy an unmet market need. Corporate employees are typically responsible for the planning and completion of every stage and the ensuing transformation of the respective method outputs from one state of knowledge to another. They are also responsible for fulfilling a profit obligation to private owners or public shareholders. Government agencies and their funded programs hold a similar obligation to their taxpayers – who are in essence shareholders – to produce effective products or services resulting in socio-economic benefit.

As such, achieving technology-based innovations capable of generating both social and economic impacts is business as usual for successful corporations. However, accomplishing the same ends through the convoluted path of research and development programs sponsored by government and conducted in non-corporate environments is much more complex. It frequently requires collaboration and investment from different categories of stakeholders [53], each of which operates in its own context with differing value systems.

**Figure 2:**
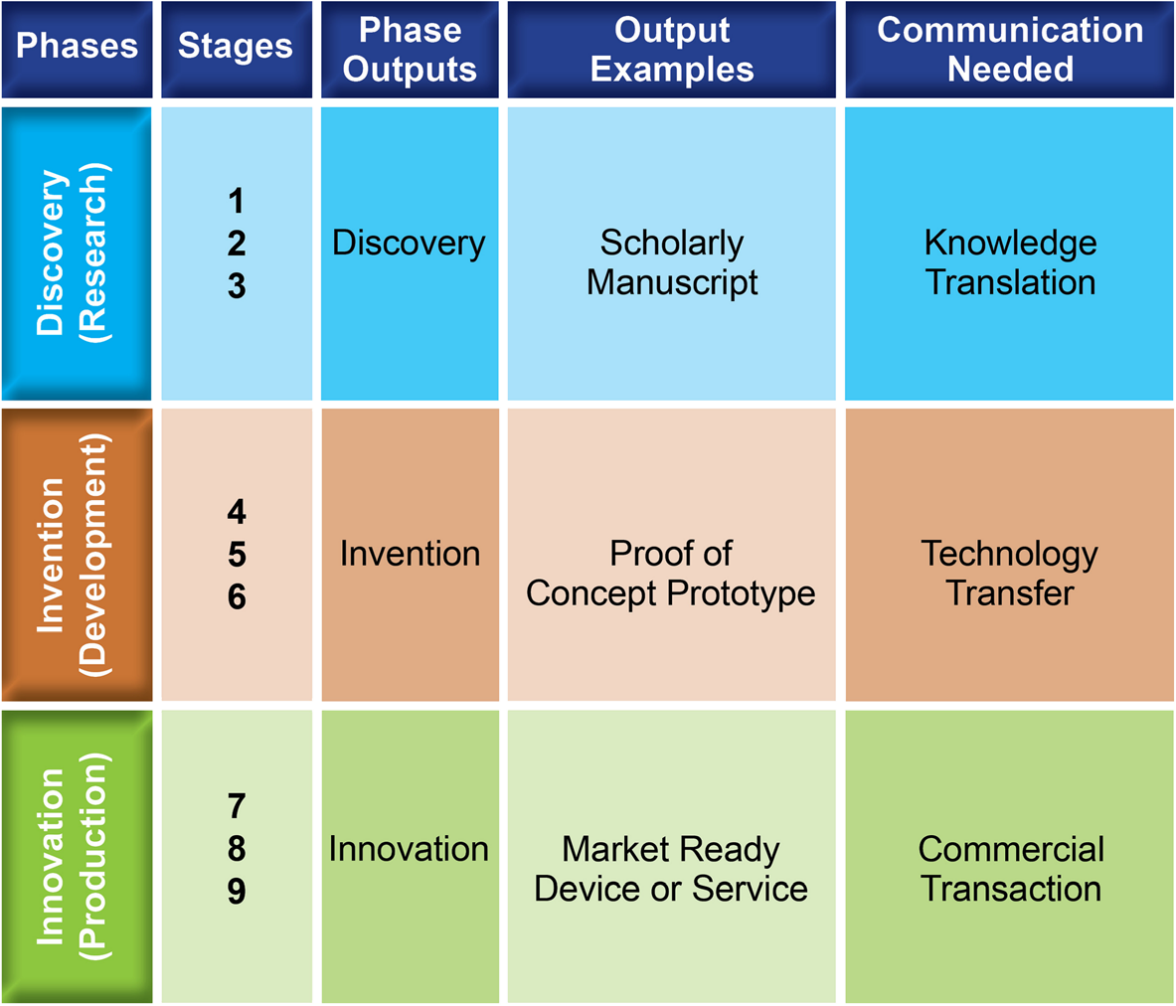
NtK framework, outputs, and modes of communication.

Being mindful that knowledge exists in three different states, the progression of knowledge from method to method and from state to state requires further understanding. As shown in Figure 2, the successful communication of conceptual discoveries – typically in the form of scholarly manuscripts – from scientific researchers to users involves a strategy of tailoring and targeting the message, currently called knowledge translation (KT). All of the elements associated with the Knowledge to Action approach [38] are appropriate for conceptual discoveries. Publications are considered intellectual property and fall under the legal protection of copyright law.

Prototype inventions are treated differently from conceptual discoveries. Ownership is controlled by patents and exchanged through patent assignment. This exchange from inventor to application manager involves a different strategy called technology transfer (TT). The technology transfer process provides legal standing to the invention users, typically corporations, who then invest their internal resources and risk their future viability on the commercial success of an envisioned product based on the invention.

Following similar reasoning, the sale and purchase of products in the marketplace is really an exchange of ownership from the manufacturer to the consumer. It represents a third strategy called commercial transaction (CT), where information about market ready devices and services is communicated from a manufacturer to retailers and/or to potential customers. This commercial market mechanism is where the incentives of supply meet the incentives of demand and thereby generate beneficial social and economic impacts. The beneficial societal impact is new functional utility for the end customer, while the beneficial economic impact is financial returns to the manufacturer and other stakeholders in the value chain.

The economic activity surrounding the entire technological innovation process creates new net wealth for the host actors, organizations and nations. This new net wealth is shared with the government through tax revenue, which is then re-distributed to all sectors through grants, contracts, entitlements, programs and services. An expanded discussion of the three forms of knowledge communication (KT, TT, and CT), and a case example are provided in Additional file 2.

By applying the KT and TT strategies outlined by the NtK, academic researchers are in effect becoming knowledge brokers for their own material. Researchers who accept public monies through programs intending to generate technological innovations with beneficial socio-economic impacts, must carry the responsibility of ensuring that the relevance of their project output is maintained throughout a research project, thereby eliminating many potential barriers to successful transfer. There is a considerable additional time requirement imposed upon successful knowledge brokers, so it is incumbent upon government programs and officials to clearly communicate the expectations for these innovation programs, and differentiate them from the traditional research mission of university scholars [36].

### Theoretical implications

The NtK offers an evidence-based starting point for the formulation of practical technological innovation policies, which increases the relevance of outputs from publicly funded research projects to industry and society alike. However, many questions remain to be answered regarding the NtK’s utility. For example, what challenges will NtK model users experience? Is the NtK applicable to incremental as well as radical technology development? In what instances can steps be eliminated to speed time to market? These questions provide the foundation for future work to test and validate the use of the NtK in practice.

### Summary

Government programs have an obligation to ensure that public resources allocated to generate beneficial socio-economic impacts are both rigorous in methodology and relevant to the intended impacts. This is particularly important for government sponsored research and development activity intended to generate technological innovations. The desired innovations are expected to improve domestic quality of life while helping to compete in a global economy. These are pressing issues, yet the predominant linear model of innovation has long trivialized the innovation process by relying on assumptions and serendipity, while eschewing explication and planning.

Scholarly studies and industrial practices addressing technological innovation have recognized the importance of considering the full continuum of required activities prior to implementing an innovation project. They have also confirmed the logic and efficiency of initiating product or service oriented interventions from the perspective of the marketplace. Once a program is initiated and projects are funded, project managers have an obligation to ensure that they are good stewards of the knowledge created through the upstream activities of scientific research, to preserve the value of that knowledge in the context of the downstream engineering development and industrial production stakeholders. These stakeholders, in turn, must ensure that the innovative product or service is valued in the context of the target customers who acquire and use them. The application of established best practices in methods and metrics will ensure the efficient delivery of effective innovations while providing return to all stakeholders from the investment of public resources. This is good business and good public policy.

The Need to Knowledge Model spans the entire technological innovation process and provides an operational-level guide to the stages, steps and activities. The acknowledgement of three related methods, the presence of knowledge outputs in three states, the values of all relevant stakeholders, and the market orientation all contribute to a clearer understanding of the technological innovation process. The NtK Model should be useful for planning, implementing, managing and evaluating programs and projects intended to generate technology-based innovations with beneficial socio-economic impacts.

## Competing interests

The authors declare that they have no competing interests.

## Authors’ contributions

JLF led the project design, data gathering, and reporting activities that established and validated the NtK Model. JPL conceptualized the NtK Model and contributed to data gathering activities. MML participated in data gathering and led the data analysis that validated the NtK model. All authors contributed content to this manuscript and participated in editing. All authors read and approved the final manuscript.

## Supplementary Material

Additional file 1 **Accessing the NtK Model – provides information on how to find the NtK Model on the KT4TT website.** Describes the game board and tabular versions of the NtK Model [39-43]. Click here for file

Additional file 2 **The critical role of six key stakeholder groups – provides descriptions and examples of six stakeholder groups who should be considered when generating and transferring new knowledge.** Includes a discussion of three mechanisms used to transmit knowledge between stakeholder groups- knowledge translation, technology transfer, and commercial transaction [31,38,52]. Click here for file
