# Supplementary material for: Need to Knowledge (NtK) Model: an evidence-based framework for generating technological innovations with socio-economic impacts
Source: Implement Sci. 2013 Feb 15;8:21. doi: 10.1186/1748-5908-8-21 (PMC3598477; doi:10.1186/1748-5908-8-21)
Supplement: Additional file 1 — Accessing the NtK Model – provides information on how to find the NtK Model on the KT4TT website. Describes the game board and tabular versions of the NtK Model [39-43]. [file 1748-5908-8-21-S1.pdf]

Additional File 1

Open access to the Need to Knowledge (NtK) Model

The Need to Knowledge Model is constructed as an online database, which is fully accessible through a website [39]. Users can view the NtK Model in one of two formats: 1) As an interactive Flash game board, or 2) As a tabular text-based version. Users navigate through both versions via hyperlinks.

NtK Model's game board version - The game board version of the NtK Model applies graphics and color to clearly differentiate the three phases of research, development and production. As discussed throughout this paper, the separation of research and development is purposeful because these two phases are commonly coupled as "R&D," despite the fact that their respective activities and outputs are not interchangeable. A segmented path connects the three phases on the game board, and advancing along the path moves the user through the stages and steps required to transform knowledge from one state to another. The knowledge to action options are embedded at each point of phase output, to guide opportunities for communication with various stakeholder groups who can move these project outputs forward through the innovation process. In the context of knowledge to action, this is considered instrumental knowledge use. In any case, knowledge outputs should be communicated externally and added to the well-spring of knowledge because they could also be applied through conceptual or strategic knowledge use [40].

NtK Model's tabular version – The tabular version is fully accessible to all users and offers more detail than the game board version [41,42]. The NtK Model's table is structured in three columns, each providing the most general information at the top level, with hyperlinks to multiple layers of information related to each stage and step underneath. The left hand column

names the stages and gates, offers links to case examples describing each stage's activity, and provides links to the supporting evidence gathered in the literature review. The right hand column offers tips for step completion, as well as links to supporting evidence when available. The middle column contains the most detail, including names of steps, with links to supporting evidence. In addition to relevant excerpts, supporting evidence for steps contains information pertaining to tools that can be used to complete each step. The supporting evidence also contains tools for performing specific scientific, engineering or marketing analysis, including descriptions, requirements and utility of each analytic tool.

In both the game board and table versions of the NtK Model, KT elements are embedded throughout, and also specifically detailed following the generation of knowledge in each of its three forms. The table version offers links to modified versions of the KTA diagram that mirror the KTA steps printed in the table and on the game board. The diagrams are supplemented by KTA tables offering additional suggestions related to completion of KT activities.

The NtK Model's underlying database enables users to perform customized searches of the supporting evidence drawn from academic and industry literature [43]. The search capabilities allow users to scour the supporting evidence excerpts with keywords. Users can tailor their searches by selecting the sector setting and/or stakeholder affiliation to which they want the search results to apply. The searches can return either listings of related citations or listings of excerpts that provide links to full citations. The search results provide further information regarding the rationale and activities to be considered when undertaking each stage or step. All NtK Model content is freely available, such that other interested individuals can perform their own unique analysis on the content.
