# Supplementary material for: Need to Knowledge (NtK) Model: an evidence-based framework for generating technological innovations with socio-economic impacts
Source: Implement Sci. 2013 Feb 15;8:21. doi: 10.1186/1748-5908-8-21 (PMC3598477; doi:10.1186/1748-5908-8-21)
Supplement: Additional file 2 — The critical role of six key stakeholder groups – provides descriptions and examples of six stakeholder groups who should be considered when generating and transferring new knowledge. Includes a discussion of three mechanisms used to transmit knowledge between stakeholder groups- knowledge translation, technology transfer, and commercial transaction [31,38,52]. [file 1748-5908-8-21-S2.pdf]

Additional File 2

The critical role of six key stakeholder groups

Generically, the six stakeholder groups can be classified as shown in Table 1, and then defined more specifically to identify the participants in the context of any particular innovation project. As shown in Table 1, the generic stakeholder descriptions (column 2) can be specifically defined in the example of the automated jar opener project (column 3).

Table 1: Six Stakeholder Groups

**Group Name**	**Generic Description**	**Tailored Description- Jar Opener Example**
End Customers	Persons who pay (directly or through a third party) to acquire and consume technology-based devices and services in the context of their daily lives.	Users- Home and professional cooks, older adults, and people with grip and hand-strength limitations.<br>Buyers- Home and professional cooks, adult children of older adults.
Manufacturers	Persons working in industry who bring products to market and who are sustained by the revenue generated.	Corporate partner.

Table 1 (continued): Six Stakeholder Groups

**Group Name**	**Generic Description**	**Tailored Description- Jar Opener Example**
Practitioners	Persons who are a source of input for the design and implementation of technology-based innovations.	Ergonomics experts who consulted with broker on functional requirements of device.
Academic Researchers & Engineers	Scientists and engineers who generate research discoveries and development inventions, and are sustained by the flow of public funds through grants, contracts and public agencies.	Persons employed by the knowledge broker's R&D center, who discovered the need. Researcher initiated efficacy study during Stage 9.
Knowledge Brokers	Enterprises engaged in communicating, protecting or negotiating the exchange of knowledge in any state.	Knowledge broker who guided KT and TT processes.
Policy Officials	Elected and appointed officials who establish the guidelines for government programs and who oversee the implementation of these policies in programs.	Corporation and Government program sponsors allocated funding and received evidence of impact via efficacy study.

Individuals and organizations represented by these stakeholder groups may all have a role in facilitating or obstructing progress along the innovation pathway. Further, each group's contributions vary across the phases and stages. For example, end customers and expert practitioners can both represent the target markets for which a technological innovation is being generated. These groups should be continuously engaged as opportunities to benefit from their perspectives arise throughout the entire process. As shown in the jar opener example, consumers were included in need identification (Stage 1), solution scoping (Stage 2), alpha and beta focus groups (Stages 5 & 6), price point development (Stage 7), launch testing (Stage 8), and post-launch evaluation (Stage 9). Academic professionals may be heavily involved in early stages, but their involvement may end if they successfully convey their expertise to other stakeholders sectors during the KT and TT processes. In the jar opener case, academic engineers and clinicians were primarily involved in the early stage need identification. Their role typically diminishes in the industrial production phase once their expertise has been absorbed within the partner corporation. However, academic scientists established an additional role for themselves in the jar opener project by initiating an efficacy study to determine the utility of the product to people with disabilities in their home settings (Stage 9) [52].

Knowledge brokers are typically involved in the transition of knowledge from one phase or stakeholder to another. An R&D center can act as their own broker depending on their level of familiarity with business practices. Properly planned projects utilize brokers throughout a project, rather than after the outputs are generated. Their inclusion helps to ensure that proper attention is given to the protection of intellectual property, and the appropriate strategy for communicating knowledge in any state. In the jar-opener case, the broker was a part of an R&D

center. However, R&D centers often rely on their University's technology transfer offices to fulfill this role.

Policy Officials may not be directly involved in innovation projects, but instead provide the resources and infrastructure which determines what innovation programs should exist, how much funding should be allocated to them, and how their performance will be evaluated in the context of the articulated social and economic goals [31]. In the jar opener example, the actions of policy makers provided the initial funding for the project. These policy officials received the efficacy study data to demonstrate the project's contribution to socio-economic goals, as justification for this technological innovation program.

The managers of technological innovation projects should be mindful of all of these stakeholders from the earliest point of identifying a problem and planning a technology-based solution. These external stakeholders may be critical to progress at any point, and their individual or collective opinions may determine whether or not a project progresses to the next stage or phase. Incorporating their perspectives – both positive and negative – helps increase the odds that a project will be successful. Conversely, failing to account for any stakeholder group may raise an insurmountable barrier to progress or eventual market success.

The NtK Model includes communicating knowledge outputs between stakeholders

The PDMA's best practices for new product development assume that the technological innovation process typically all happens within one single organization. Further, the PDMA's best practices are silent on the changing state of knowledge as it progresses from a state of conceptual discovery, through a state of prototype invention, and out to a state of commercial innovation. Under the conditions of open innovation – or where the government intervenes in

the absence of sufficient market incentives -- the technological innovation process needs to also account for the communication, exchange and control of knowledge between different stakeholders, organizations and sectors. It should also recognize that the strategies involved will vary depending on the state the knowledge is in.

To ensure the NtK Model accounts for these factors, Lane & Flagg [31] adopted and then adapted the Knowledge to Action (KTA) Model, articulated by the Canadian Institutes for Health Research [38]. The KTA Model offered a flexible structure to apply in the context of technology-based innovation. It was originally intended to address a global need to increase uptake and use of scientific research discoveries by practitioners, so it assumed the conduct of scientific research from the outset, and assumed in advance that an output from scientific research was relevant to the target audience. Our adaptation was to expand the KTA model's front end to eliminate these assumptions and instead begin each project with the identification of a need and the potential for technology-based knowledge to address it. With that front end in place, we considered how each methodological phase would generate knowledge in a different state, and how those different knowledge states would necessarily influence any strategy for exchanging knowledge between stakeholders.

An example of communicating knowledge between stakeholders

Consequently, the NtK Model contains a link from each knowledge output state (discovery, prototype product), to a strategy for effectively communicating that knowledge to targeted stakeholder audiences. Figure 3 depicts the primary elements of the knowledge translation strategy used to communicate discovery outputs to stakeholders such that they can become inputs to the development phase. These knowledge to action strategies are also linked to KT

tables that summarize the considerations for communicating knowledge to each of the generic stakeholder groups, along with descriptions of their potential outcomes. As with the NtK's stages and steps, the generic content can be readily tailored for inclusion in any individual innovation project.

Figure 3: Knowledge Translation Activities

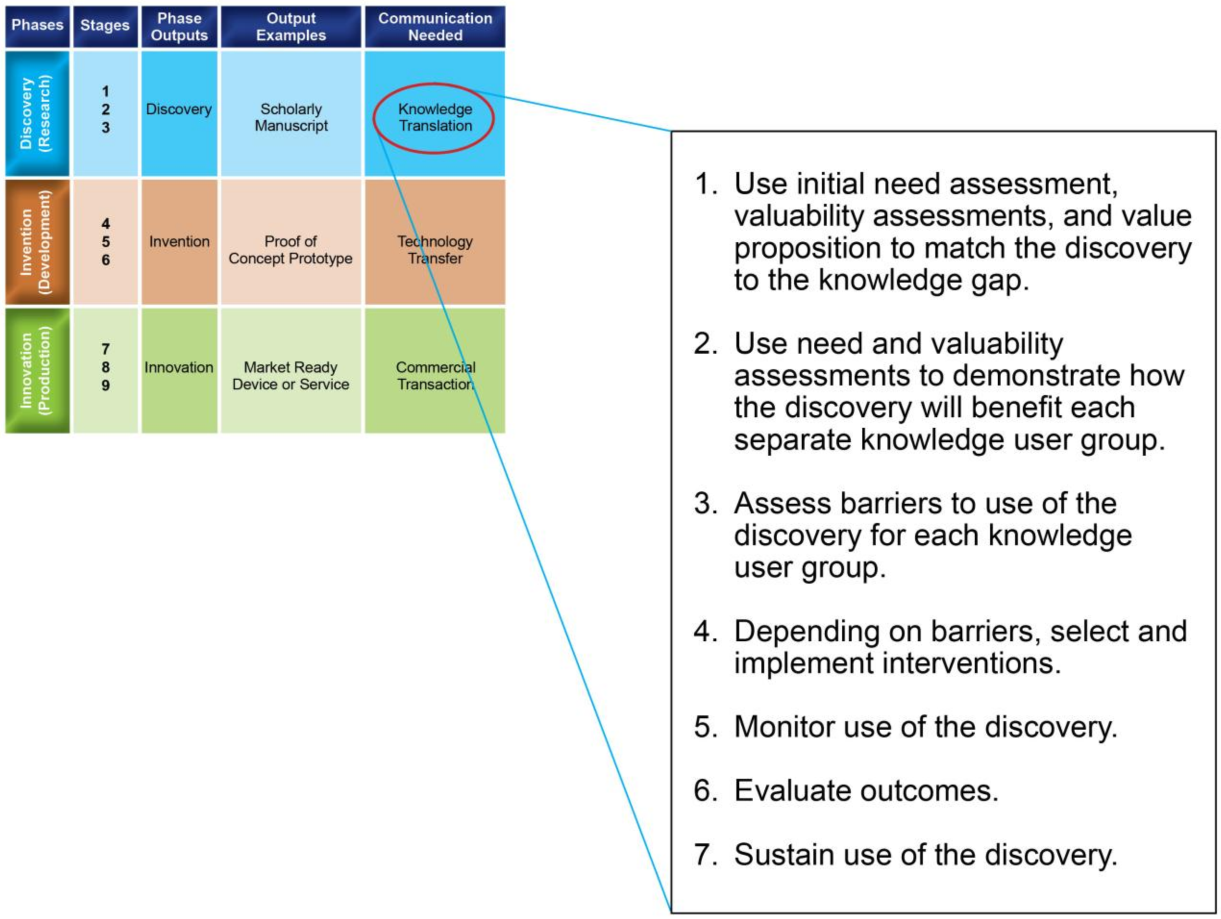

The jar opener example provides an effective illustration of how knowledge can be communicated between stakeholder groups to successfully advance through and between the three phases of the innovation process.

Knowledge translation of the conceptual discovery - KT activities in the research phase of the jar opener example included the broker's communication with the potential manufacturing partner to validate the size of the market need and the feasibility of the proposed solution, both in the context of a competitive opportunity for the company. This required the broker to translate existing knowledge into a business case using terms the company would understand and value.

Technology transfer of the prototype invention - The knowledge broker's early efforts resulted in the corporation's decision to collaboratively pursue commercialization with the Broker. When engineering activities progressed to the point of design specifications, multiple rounds of communication r were necessary for the broker to communicate consumer feedback to the corporation's development team.

Commercial transaction for the commercial innovation - One last round of communication between stakeholders occurred during the production phase. Based on positive marketing feedback obtained earlier in the process, the corporation was preparing for a product launch to coincide with the fourth quarter holiday shopping season. The corporation now shifted its focus from early stage production activities to communicating the product's value to the target customers through the creation of informational product packaging as well as a major marketing campaign.

Given the varied ways in which knowledge is communicated at different points in the innovation continuum, the NtK Model needed to represent communication as explicitly as it represented activity within each of the three methods. The modified KTA cycles representing Knowledge Translation, Technology Transfer, and Commercial Transactions, combined with the Stakeholder tables, provide the required level of detail.
